# Nanotechnology, Green Synthesis and Biological Activity Application of Zinc Oxide Nanoparticles Incorporated Argemone Mxicana Leaf Extract

**DOI:** 10.3390/molecules27051545

**Published:** 2022-02-25

**Authors:** Maheswari Chinnapaiyan, Yashika Selvam, Fatma Bassyouni, Mathammal Ramu, Chandrasekar Sakkaraiveeranan, Aravindan Samickannian, Gobi Govindan, Matheswaran Palaniswamy, Uthrakumar Ramamurthy, Mohamed Abdel-Rehim

**Affiliations:** 1 Department of Mathematics, Muthayammal College of Arts and Science, Rasipuram 637408, Tamil Nadu, India; mahi2gobi@gmail.com (M.C.); mathammalphy18@gmail.com (M.R.); 2 PG & Research Department of Mathematics, Arignar Anna Government Arts College, Namakkal 637002, Tamil Nadu, India; chandrumat@gmail.com; 3 Department of Physics, Sri Sarada College for Women (Autonomous), Salem 636016, Tamil Nadu, India; yashikaphy@gmail.com; 4 Department of Natural and Microbial Products, National Research Center, Cairo 12662, Egypt; 5 Department of Physics, Government Arts College (Autonomous), Salem 636007, Tamil Nadu, India; aravindan.sp@gmail.com (A.S.); gobi.rect@gmail.com (G.G.); ukloyola@gmail.com (U.R.); 6 Department of Chemistry, Government Arts College (Autonomous), Salem 636007, Tamil Nadu, India; matheshkutty77@gmail.com; 7 Department of Materials and Nanophysics, KTH Royal Institute of Technology, SE-11419 Stockholm, Sweden; mohamed.astra@gmail.com

**Keywords:** nanoparticles ZnO NPs, green biosynthesis, characterization techniques, antibacterial and antifungal activities, antioxidant activity

## Abstract

Nanomaterial is a rapidly growing area that is used to create a variety of new materials and nanotechnology applications from medical, pharmaceuticals, chemical, mechanical, electronics and several environmental industries including physical, chemical and biological nanoparticles are very important in our daily life. Nanoparticles with leaf extract from the healthy plant are important in the area of research using biosynthesis methods. Because of it’s used as an environmentally ecofriendly, other than traditional physical and chemical strategies. In particular, biologically synthesized nanoparticles have become a key branch of nanotechnology. The present work presents a synthesis of zinc oxide nanoparticles using an extract from the Argemone leaf Mexicana. Biosynthetic nanoparticles are characterized by X-ray diffraction (XRD), Ultraviolet visible (UV-vis) spectroscopy analysis, a Fourier Transform Infrared Spectroscopy analysis (FTIR) and a scanning electron microcopy (SEM), X-ray analysis with dispersive energy (EDAX). XRD is used to examine the crystalline size of zinc oxide nanoparticles. The FTIR test consists in providing evidence of the presence of targeted teams. UV is used for optical properties and calculates the energy of the bandwidth slot. The scanning microscope emission reveals the morphology of the surface and the energy dispersive X-ray analysis confirms the basic composition of zinc oxide nanoparticles. It is found that zinc nanoparticles are capable of achieving high anti-fungal efficacy and therefore have a high potential antimicrobial activity of ZnO NPs, like antibacterial and high antioxidant. Zinc Oxide nanoparticles from the Argemone Mexicana leaf extract have several antimicrobial applications, such as medical specialty, cosmetics, food, biotechnology, nano medicine and drug delivery system. ZnO nanoparticles are important because they provide many practical applications in industry. The most important use of nanoparticles of ZnO would be strong antibacterial and antioxidant activity with a simple and efficient biosynthesis method may be used for future work applications.

## 1. Introduction

Nanotechnology is the most important dynamic exploration regions in the current of material science. Nanoparticles have entirely new and more developed research based on specific factors such as size, dispersion and morphology. Nanotechnology technique is an area that develops day-to-day, and affecting all parts of the human existence [1,2,3]. Nano zinc particles have found surprising applications in the field of high molecular identification and demonstration [4], antimicrobials, therapeutics [5,6], catalytic converters [7] and miniature equipment [8]. However, there is still a requirement for application in industrial expert and a widely approach to the integration of zinc nanoparticles. Numerous techniques applied, for example, the combination of zinc nanoparticles, the reaction of substances and the reaction of compounds changing micelles, the extensively used of zinc oxide in radiation [9,10,11,12], the electrochemical, sonochemical, microwave [13,14,15] and green science applications [16,17,18].

Natural techniques have been prepared for a “greener Union” of nanoparticles and have been shown to be more efficient due to their slower energy, giving better focus product and control over the development and reliability of valuable compounds economically, scientifically and environmentally valuable compounds and materials in nanostructure form.

This has strengthened of an important of nanotechnology pathway, which includes better control of the shape and size of various nano-applications. The uses in microscopic and micro sheet applications have increased as well as used as antimicrobial and antifungal agents in addition to nano medicine and as catalysts [19,20,21,22]. Zinc nanoparticles offer numerous environmental benefits and similarities of medicines to other natural uses. The green compound is improving for substances and real techniques are increasing, eco-friendly, accurately estimated in large quantities and there is no convincing reason to use high pressures, energy, temperature and synthesis. It has been known for a long time that zinc is caused by the presence of inhibitory organisms used in the clinical and modern system. Nanopharmaceutical, including drug and medical nanomaterial, and useful nanoparticles used as anti-microbial agents or functional nanostructures used for the detection of biomarkers, nano chips, nanoparticles are very important in our life in the application of nanotechnologies [23,24]. The main use of zinc nanoparticles and zinc oxides is in clinical and biomedical applications as well as the supply of medicines, drug delivery and biosensor [25].

**Figure 1:**
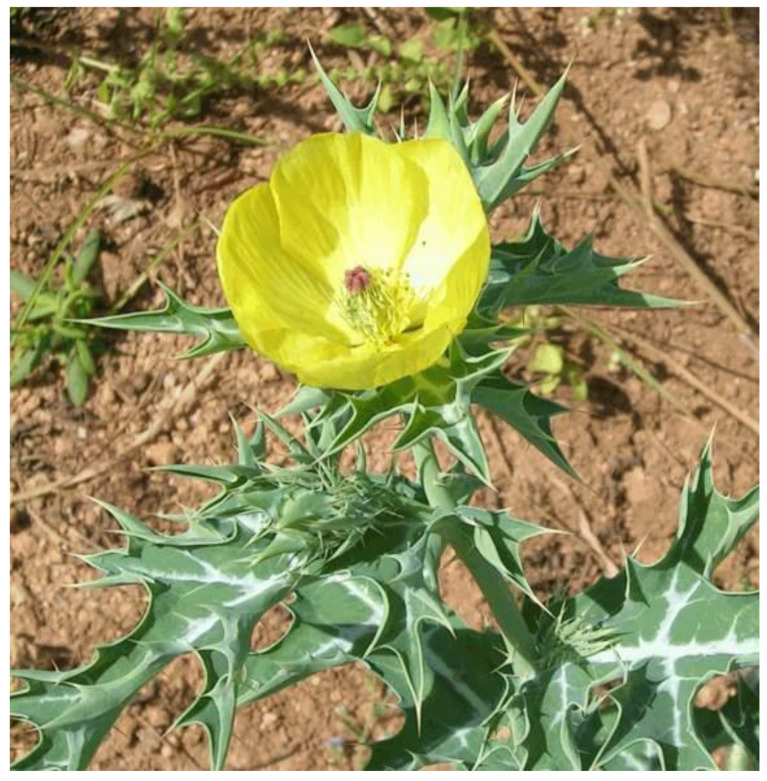
Image of Argemone Mexicana Plant.

Argemone Mexicana is a medical plant is shown in Figure 1 and can be used to treat various diseases as fever, malaria, jaundice, vagina, skin diseases. It is widely distributed in India and grown in the garden and on the road side, wild boar in the hills of north-western India. Argemone is botanically classified with two Latin names, namely Mexican mushroom and satranishi. The species of Argemone Mexicana Linn is subject to the family Papaveracea and the name of the species in Tamil is Bramatandu. The plant is used in various parts of the world to treat several conditions, including cancer, osnowiec, skin disease, inflammation, rheumatism, jaundice, tritzies, microbiological infections and malaria. Interestingly, the plant is a source of various kinds of chemical ingredients, although alkaloids are mostly rich. Therefore, this work reveals the synthesis and examines the properties of zinc oxide nanoparticles, using the extract from the leaves of Argemone Mexicana as cheaper natural products, particularly suitable for the medicinal compounds [26,27].

In the present study, we report a combination of nanoparticles of zinc and reducing the zinc particles present in zinc acetic acid derivative arrangement by the fluid extraction of the AML. These nanoparticles have been discovered to be profoundly their leaves and are deeply active against various pathogenic microscopic organisms as antimicrobial and antioxidant agents.

## 2. Results and Discussion

### 2.1. X-ray Diffraction Analysis

**Figure 2:**
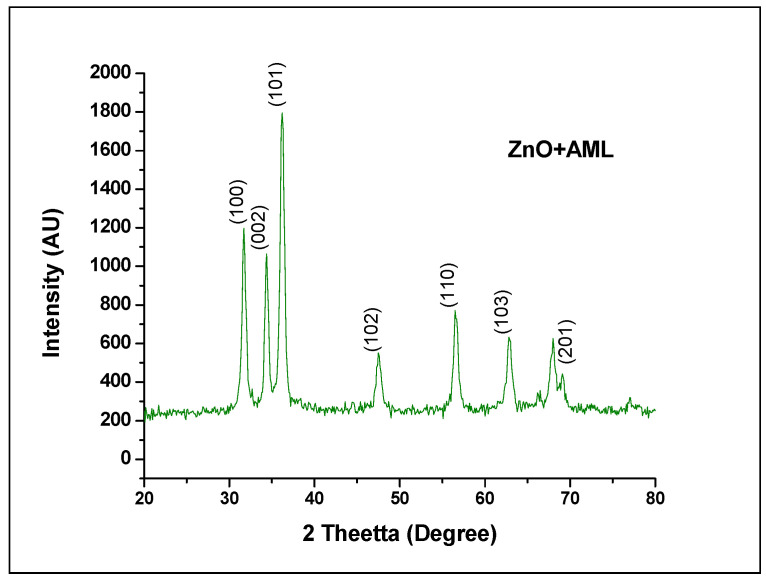
XRD analyses of zinc oxide nanoparticles (ZNP) with indexed crystalline peaks.

Analysis of the X-ray diffraction (XRD) helps to ensure that zinc oxide nanoparticles are formed from the extract solution of the leaves of ZnO and Argemone Mexicana (AML). The XRD zinc powder pattern is shown in (Figure 2), showing the diffraction peaks of the ZnO crystal structure in specific phases. The acute peaks of the XRD pattern indicate the well-organized crystalline nature of the yield product. The data are some specific crystalline peaks that are well agreed with the JCPDS Charter No 89-1397 the emergence of the ZnO nano-structure has been ensured. The diffraction mesh planes belong to crystalline peaks, as shown in (Figure 2), are indexed as (100), (002), (101), (102), (110), (103), and (202) in turn confirmed the hexagonal structure of ZnO nanoparticles. This formula is consistent with the standard peak values displayed by the International Diffractive Data Center (IDDC). The average size of crystalline zinc nano particles was estimated on the basis of data from the formation of crystalline peaks. The observed XRD test results shall examine the well-organized crystalline structure of the yield product as a result of the use of the raw processing product including the Argemonac Mexicana leaf extract (AML). It proves the product that is being developed materials, the results showed in Figure 2 and Table 1.

(1) $$D=\frac{k\lambda }{\beta \;\mathrm{Cos}\theta \;}\;\mathrm{nm}$$

where,

k = Scherrer’s constant (0.9)
λ = Wavelength of X-ray (1.54 × 10^−10^ m)
β = Full Width Half Maximum
θ = Bragg’s angle

The particle size of zinc oxide Nano particles is 1.46 Nm

### 2.2. FTIR Analysis

**Figure 3:**
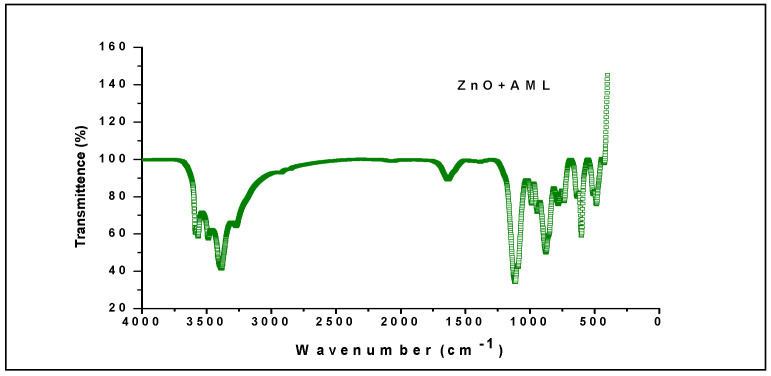
FTIR analyses of zinc oxide nanoparticles (ZNP).

Infrared spectroscopy analysis with Fourier transformation helps to investigate the functional behavior of sample peaks. It is well known that the optical response of molecular species in the form of vibrations followed by the absorption of the IR signal is recorded as a spectral analysis, as shown in Figure 3. The elemental components of zinc oxide nanoparticles reacted well to the IR signal by displaying the absorbance. 

The FTIR spectrum is shown in Figure 3, the peaks observed at distances of 1650 and 3353 cm^−1^ contribute to bending and stretching the moisture vibrations present in the which is seen in spectral peak in the form of absorbance assigned to leaf extract, and was observed in the region of 3196, 1650, 1102 and 871 cm^−1^. The exhibited absorption bands, especially in the corresponding rang 3353, 3196 cm^−1^ (phenol O-H), 1626 cm^−1^ of (C=C), 1650 cm^−1^ for (C=N), 1102 cm^−1^ for (C-O) and 871 (C=C bending), proves the existence of synthetic and atmospheric solution residue Table 2.

### 2.3. Scanning Electron Microscopic Analysis (SEM)

**Figure 4:**
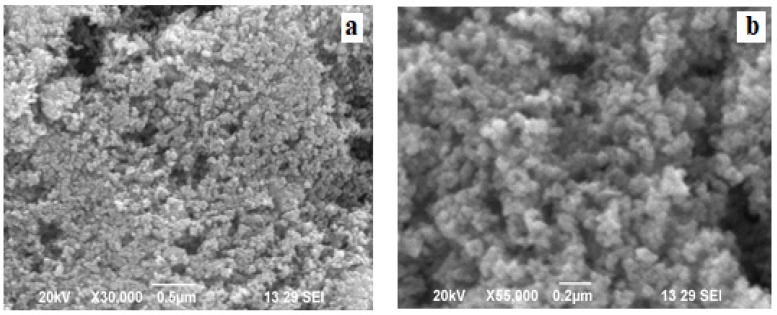
(**a**,**b**) SEM micrographs of ZnO nanoparticles with AML.

Electron microscopy of microscopic analysis SEM used to examine the shape, structure and size of synthesized ZNP as shown in Figure 4a,b. The results of the SEM show that, at different magnification, nanoparticles are of size and shape. As can be seen from the use of zinc acetate as a result, the zinc oxide molecules grow slowly and form small spherical structures and accumulate like spheres. On the other hand, using zinc nitrate as a spring, spherical ZNP is formed and nanoparticles grow and accumulate to form flower-shaped beams. This agglomeration is due to the polarity and electrostatic attraction of ZnO nanoparticles. Similar observations were documented by Divya et al. [28].in the current study on the green synthesis of ZNP by Argemone Mexicana, the leaf in water extract and zinc acetate as precursors, the shapes of nanoparticles were like flower bunches. As seen, the image indicates that the particles were present in both diffuse and agglomerated mono with approximately spherical morphology. Such variations in particle size and shape distribution can be explained by the chemical structures of the various components contained in plant extracts Figure 4a,b.

### 2.4. Energy-Dispersive X-ray Spectroscopy

**Figure 5:**
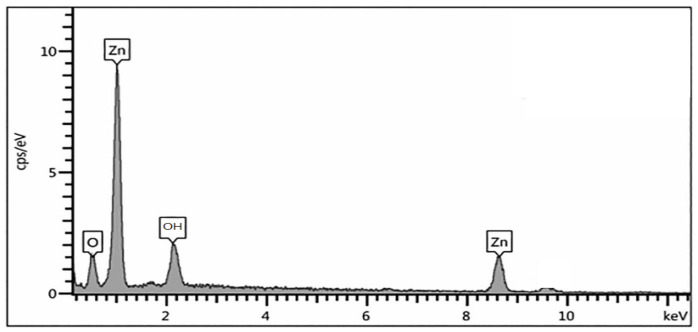
EDAX Spectrum of Zno Nps.

The analysis of the elemental composition of the ZNP from the EDAX graph, as shown in Figure 5, reveals that the elemental components of the samples are present on the surface. The EDAX spectrum shows that the required Zn and O phase is present in the samples and confirms high purity for synthesized ZNP. The hypothetical expected percentage of stoichiometric is 32.05% and 34.58% respectively. The analysis of EDAX in our study shows similar results for both synthesized nano particles, while in the previous study the elemental composition of zinc and oxygen was 60.16% and 12.87% [29], respectively. The existence of OH components records the rewinding components existing on the sample surface. Since the EDAX analysis observed in the SEM analysis, the particles that appear in the SEM images are provided as components of zinc nanoparticles. As expected, the percentage of Zn above the oxygen content is dominant and confirms that the prepared samples are a product of zinc oxide nanoparticles prepared under the influence of an AML extract (Figure 5 and Table 3).

### 2.5. Ultraviolet—Visible (UV-Vis) Spectroscopy

**Figure 6:**
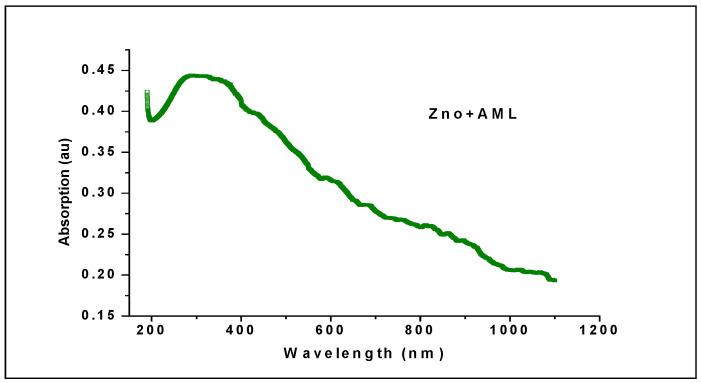
UV-Visible Spectrum of ZnO Nps.

Spectral analyzes used to test the performance of elemental components exist in the sample. Ultra Violet—visible (UV-vis.) Spectroscopy analysis is a visible observation that can be used to examine the electronic configuration of the root structure. It is well known that ZnO has an absorption edge in the wavelength range 200 to 400 nm. UV-vis observed. The spectrum shows the peak absorption around the wavelength of 377 nm and records the adsorption of the electronic ZnO bandwidth gap. This result is a good agreement with the values eV and will investigate the cause of the removal of the Valencia electron. The estimated value of the gap in the electronic energy band is 3.2 eV and ensured the creation of a crystalline structure of Nano ZnO with a reduced value of the band gap. The ZnO bandwidth value is approximately 3.57 eV, which is estimated in the work. Results show a reduction in the nano-scale ZnO particle size. The present value clearly showed a significant decrease in the bandwidth gap as 3.2 eV as shown in Figure 6.

### 2.6. Antibacterial Activity

From the anti-microbial test, the activity of synthetic extracts of ZnO NPs and leaves gave very good inhibits growth of both *E. coli and S.aureus* and the results are summarized in the Table 4. Leaf extract as well as high ZnO NPs concentration gives high activity. The *E. coli* organism inhibition zone is larger than zone *S.aureus* with a ZnO NPs concentration of 75 µL and 100 µL. The anti-microbial activity of the synthesized ZnO NPs is expressed as a potential activity than pure leaf extract. Since zinc ions exposed to leaf extract have a greater for action. The synthetic ZnO NPs shows the maximum inhibitory effect against both Gram positive bacteria and Gram-negative bacteria (*E. coli and Staphylococcus aureus*). The anti-microbial activity of ZnO NPs has a standard *E. coli* inhibition zone of 8 to 11 mm and *Staphylococcus aureus* from 7 mm inhibition zone. The respective concentration is 75 µL and 100 µL for both organisms.

### 2.7. Antifungal Activity

The anti-fungal properties of the ZnO NPS, the results indicate that zinc nanoparticles have good anti-fungal activity against the *Aspergillus fumigate* micro-organism. It is confirmed that zinc nanoparticles are capable of achieving high anti-fungal efficacy and therefore have a high potential activity of ZnO NPs, which is clearly visible in the inhibition zone by the growth of the tested micro-organisms in Table 5.

**Figure 7:**
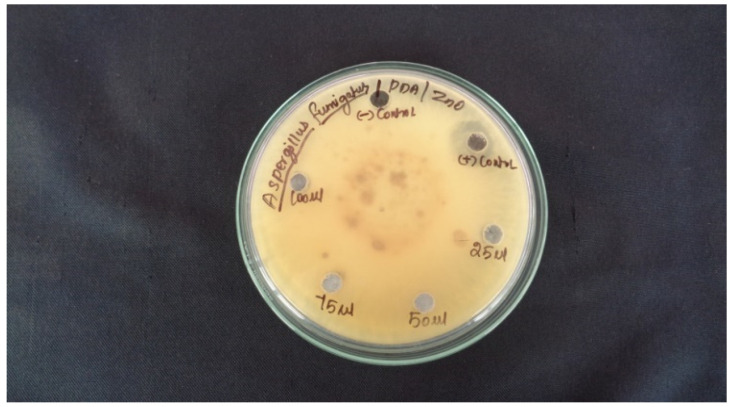
Antibacterial activity of ZnO Nps and Leaf extract.

Figure 7 shows the action of the synthetic ZnO NPs from the plant extract Argemone Mexicana against *Aspergillus fumiga*. 

### 2.8. Antioxidant Activity

**Figure 8:**
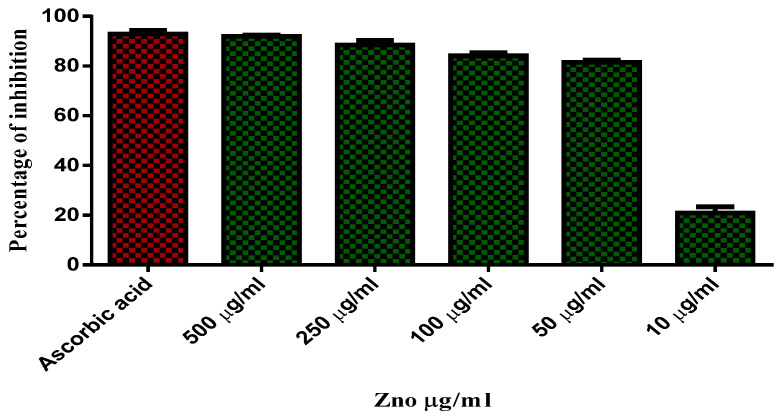
The Percentage inhibition concentration of Ascorbic acid.

The results are shown in Table 6. The relative braking zone of the sample is shown in Figure 8. The sample exhibited significant activity at the lowest concentration of IC_50_. The color changes from purple to yellow. Ascorbic acid has been used as a standard with an IC_50_ value of 92 ± 0.1 µg/mL. The absorption values of the IC_50_ ascorbic acid are shown in Table 7. The relative braking zone of the sample is shown in Figure 8. A lower absorbance value and IC_50_ indicated that NPS ZnO has anti-oxidative properties.

## 3. Materials and Methods

### 3.1. Preparation of Leaf Extract

The green and fresh leaves of Argemone Mexicana are carefully washed from tap water and double distilled water. Then 10 g of leaves were collected and ground into the leaf extract with a mortar and add 50 mL of distilled water and 20 min mixed. After 20 min, a pure leaf extract of 10 mL is collected by the beaker solutions, filtered through whatman filter paper No 1 and used.

### 3.2. Biosynthesis of Zinc Oxide Nanoparticle

The zinc acetate dihydrate was prepared by dissolving 3.8 g (CH_3_COO)_2_ Zn^+^2H_2_O in 50 mL of double distilled water and mixed for half an hour. Then 10 mL of the extract from the solution is added to the Zn acetate solution, leaving a drop by drop and stirring for two hours until nanoparticles are formed. The PH value is maintained at 12 by the addition of NaOH. Finally, the color of the solution changes from a deep-low-level green colored paste. Nanoparticles are deposited at the bottom of the beaker. The clear solution is removed and therefore the particles are thoroughly rinsed twice daily with distilled water for four days. The extract solution is then removed and the particles are collected and placed over a furnace of hot air at low temperature for 100 °C for five hours. After the sample has been transferred to silica crucible cup and muffle furnace at 400 °C for 2 h, the sample has finally been taken to the mortar and ground in a fine nano powder. These powdered nanoparticles have been used for characterization purposes (Table 8).

### 3.3. Antibacterial Activity Assay

The Nanoparticles of oxide have been active in relation to Gram positive and Gram-negative bacteria, with a high degree of 100 µg, guided by the diameter of the inhibition zone. The braking zone is detected with the leaf extract (Argemone Mexicana) and a better concentration of zinc nanoparticles (100 mg/mL). The presence of a braking zone clearly indicates the outcome of the treatment of nanoparticles of ZnO. It was noted that the concentration of nanoparticles of ZnO is increased; the expansion inhibition was also increased. The dimensions of the braking zone are completely different depending on the type of bacteria, the dimensions and the concentration of nanoparticles of ZnO [30,31].

### 3.4. Antifungal Activity Assay

The anti-fungal properties of the ZnO NPS solution of leaf extract were determined using *Aspergillus fumiga*, anti-fungal activity in diffusion assay method and its signs of growth of fungi in the diffusion assay. The action of the synthetic ZnONPs from the plant extract Argemone Mexicana against *Aspergillus fumiga* is determind. The maximum inhibition zone was observed at 5 mm for 25 µg, 3 mm for 100 µg/mL concentrations. In *Aspergillus fumigus*, the zone increases as the concentration increases [32].

### 3.5. Antioxidant Activity

The activity of radicals of synthesized nano compounds is determined on the basis of the ability to scavenge stable DPPH radicals [33]. The antioxidant capacity of zinc oxide nanoparticles at different concentrations is calculated as a percentage inhibition of absorbance. DPPH is stable, free radicals that could accept either electron or hydrogen radicals in order to become a stable molecule. The reduction of DPPH of the rhodium is determined by the decrease in its absorbance caused by the antioxidant at 517 nm. The concentration of the sample at which the percentage of inhibition reaches 50% is its IC_50_ value. The IC_50_ value is negative due to antioxidant activity because it expresses the amount of antioxidant needed to reduce the concentration of its radicals by 50%.

## 4. Conclusions

Nanotechnology is the most promising technologies of the 21st century. It is used in several medical and biological applications. A green biosynthesis of zinc oxide nanostructure (ZNP) has been successfully applied under the influence of Argemone Mexicana (AML) leaf extract. Characterization of prepared ZnO nanostructures studied using a prominent analysis such as XRD, SEM, EDAX, FTIR and UV-vis. Spectroscopic analysis, etc. the crystalline, structural, morphological and spectral properties, using the results of the crystalline studies obtained, the formation of nano-crystalline structures of ZnO could be investigated. The observed spectroscopy analysis helps to reveal the function of the elemental composition. The analysis of EDAX reveals on its surface the existence of Zn and O. The observed UV-vis analysis exhibited an absorption peak at 377 nm and provided the energy value of the ZnO nanoband gap at 3.2 eV. Biosynthetic nanoparticles of ZnO gave a strong level of anti-microbial activity against *E. coli* (negative grammatical bacteria) and *Streptococcus aureus* (positive grammatical bacteria) and a stronger antifungal activity reported in *Aspergillus fumiga*. Leaf extract as well as high ZnO NPs concentration gives high activity. It has been found that ZnO nanoparticles are synthesized to protect against bacterial and fungal pathogens, suggesting that they can be used as effective antimicrobial and antioxidant agents for commercial uses in biomedical applications and may be medical therapy for other future work and applications in industry.

## Figures and Tables

**Table 1:** Crystalline size and hkl value of observed crystalline peaks.

S.NO	2 θ Degree	d A°	FWHM (deg)	Hkl	Crystalline Size (nm)	Average Crystal Size (nm)
1.	31.737	2.820	0.56300	100	1.47129	1.461449
2.	34.379	2.608	0.54300	002	1.532493	
3.	36.215	2.480	0.60570	101	1.380564	

**Table 2:** FTIR Peak Values.

Peak Value	Chemical Bonding
871 cm^−1^	C=C bending
3196 cm^−1^	O=H Stretching
1626 cm^−1^	C=C Stretching
1650 cm^−1^	C=N Stretching
1102 cm^−1^	C-O Stretching

**Table 3:** EDAX Analysis for Zinc Nanoparticles.

Sample	At of (Zn)	At of (O)
ZNp	32.05	34.58

**Table 4:** Antibacterial activity of ZnO against *Escherichia coli* and *Staphylococcus aureus*.

Sample No	Sample Marking	Sample Concentration	Test Organisms and Zone of Inhibition in (mm)	
			*Staphylococcus* *aureus*	*E. coli*
2	Control	MHA	NA	NA
3	Leaf Extract	75 µL	NA	8 mm
4		100 µL	7 mm	11 mm

**Table 5:** Antifungal activity of ZnO nanoparticles.

Sample No	Sample Marking	Sample Concentration	Zone of Inhibition in (mm)
			*Aspergillus fumiga*
2	Control	PDA	NA
3	ZnO AML	25 µL	5 mm
4		75 µL	NA
5	Leaf Extract	50 µL	NA
6		100 µL	13 mm

**Table 6:** The Percentage inhibition of Argemone Mexicana Leaf Extract.

Sample No	Extract Concentration (μg/mL)	DPPH Antioxidant Activity		
		OD Value at 517 nm (in Triplicates)		
1	500 μg/mL	1.613	1.244	1.507
2	250 μg/mL	0.108	0.120	0.122
3	100 μg/mL	0.152	0.153	0.198
4	50 μg/mL	0.211	0.236	0.247
5	10 μg/mL	0.283	0.253	0.273
6	Control	1.189	1.148	1.116

**Table 7:** The Percentage inhibition and IC_50_ value of Ascorbic acid.

Sample No	Standard	Concentration (g/mL)	Antioxidant Activity % of Inhibition
			DPPH (510 nm)
1	Ascorbic acid used as a standard (OD value)	500 μg/mL	91.97
2		250 μg/mL	88.46
3		100 μg/mL	84.08
4		50 μg/mL	81.44
5		10 μg/mL	20.83

**Table 8:** Details of green biosynthesis experiment procedure.

Day	Materials and Methods	Process
**I**	3.8 g of Zinc acetate dihydrate+ 50 mL of distilled water	Stirred for 30 min.
	10 g leaves of Argemone maxicana+50 mL of distilled adding 60 mL of leaf extract (drop wise)	Stirred for 20 min + Filtering extract with whatmann filter paper.
	Formation of Zinc nanoparticles (deep emerald green color)	1hour of stirring (PH = 12) and then kept in room temp.
**II**	Deep emerald green precipitation was formed	Per day for Two times water changed.
**III**	Deep emerald green precipitation was formed	Per day for Two times water changed.
**IV**	Dried in hot air oven at 100 °C for Five hour	Sample transferred to silica crucible cup.
**V**	Heated in muffle furnace at 400 °C for 2 h. White precipitation was formed finally.	Grained in mortar ZnO Nano powder.

## Data Availability

All the Data is available with Authors.

## Sample Availability

Samples of the compounds are available from the authors.
